# Investigation of the dynamic bending properties of MoS_2_ thin films by interference colours

**DOI:** 10.1038/srep18441

**Published:** 2015-12-18

**Authors:** Peng Wang, Si Xiao, Xiaohong Li, Bosai Lyu, Yingbao Huang, Shubo Cheng, Han Huang, Jun He, Yongli Gao

**Affiliations:** 1 Institute of Super-microstructure and Ultrafast Process in Advanced Materials, School of Physics and Electronics, Central South University, 932 South Lushan Road, Changsha, Hunan 410083, P. R. China; 2 Department of Physics and Astronomy, University of Rochester, Rochester, New York 14627, United States

## Abstract

A non-contact method for the observation of the elastic deformation of 2D molybdenum disulfide (MoS_2_) thin films using an ordinary optical microscope is reported. A pulsed laser is used to rapidly increase the bending deformation of the MoS_2_ thin films via heating. The bending angle of the MoS_2_ thin films shows high stability, changing only 5% in forty days without external forces. However, the bending angle of the MoS_2_ thin films substantially decreases after being wetted with the volatile polar solvent tetrahydrofuran (THF), because of its low surface tension. By removing the nano-Newton scale forces on the MoS_2_ thin films, the bending angle increases significantly within 4 minutes, and this feature of the thin films shows great potential for use in the fabrication of micro-force sensors. This is the first attempt to study the mechanical properties of 2D materials by optical methods. Further utilization of industrially manufactured MoS_2_ thin films for detecting micro-force qualitatively on the basis of their excellent bending properties would significantly reduce the production costs of micro-force sensors.

Molybdenum disulfide (MoS_2_) is an emerging two-dimensional (2D) material^1^, and has attracted considerable research interest^2^,^3^. Large-scale MoS_2_ thin films can be fabricated with liquid-phase exfoliation methods^4^,^5^. MoS_2_ shows excellent elastic properties, and its Young’s modulus has been measured precisely^6^,^7^. Monolayer MoS_2_ has a smaller Young’s modulus (270 ± 100 GPa)^8^ than graphene (1000 GPa)^9^, which makes MoS_2_ a more suitable material for microdynamometers. The Young’s modulus of a monolayer MoS_2_ flake is similar to that of a bulk MoS_2_ crystal^10^, and the difference in thickness should not result in appreciable errors when fabricating an elastometer. According to the proportional relationship between the force and bending degree, micro-forces can theoretically be characterized using a MoS_2_-based elastometer. Regarding the practical sample preparation process via the dispersion method, the characteristic parameters of MoS_2_ thin films, such as their shape, are not uniform, which would exert a small influence on the elastic bending deformation properties under force. Hence, if a relatively simple and low-cost non-contact detection method with improved accuracy can be found, both the mechanical property characterization of 2D materials and the manufacture of microdynamometers based on 2D materials would benefit.

Currently, how to precisely measure forces at the nano-Newton and even pico-Newton scales is attracting a great deal of interest^11^,^12^,^13^,^14^. The principles are mainly based on electrostatic effects^12^, piezoelectric/piezoresistive effects^13^, or the elastic deformation of the microcantilever of an atomic force microscope (AFM)^14^. For instance, Rasuli *et al.* have measured the force down to the pico-Newton scale using AFM cantilevers with a known Young’s modulus^15^. At present, AFM is the most commonly used instrument used to measure nanomechanical properties^9^,^16^,^17^,^18^. Dave *et al.* have studied the nanomechanical properties of MoS_2_ and WS_2_ with an improved AFM technique for nanoindention^9^ in which force and displacement are simultaneously obtained by monitoring the deflection of a cantilever with a tip. However, the spring constant of the AFM cantilever is difficult to measure accurately, which increases the uncertainty in the measurement of micro-forces^19^,^20^. Therefore, the accuracy of the microcantilever’s spring constant is very important. In addition, a complex structure and precise instruments are required to manufacture piezoresistive and electrostatic force sensors.

In this study, a non-contact method in which only the changes of the coloured fringes are observed is reported for the determination of the elastic deformation of 2D materials (MoS_2_). The bending degrees of the MoS_2_ thin films are measured, and the error determined by AFM is less than 2%. In addition, modulations are consistent with the experimental results showing that the gap variation leads to the changes in the interference colours. Finally, we measured the nano-Newton forces on the basis of the elastic property of the MoS_2_ thin films directly with a simple optical instrument. Our findings show that micro-forces can be characterized qualitatively on the basis of the elastic bending of commercial MoS_2_ thin films, which would reduce the cost of micro-elastic dynamometers.

## Materials and Methods

The MoS_2_ crystals used in all the experiments were purchased from Tianjin Kermel Chemical Reagent Co., Ltd(Tianjin, China), as was the reduced iron powder (magnetic powder). The MoS_2_ nanosheets were fabricated with the liquid-phase exfoliation method^9^. The bulk MoS_2_ crystals were added to tetrahydrofuran (THF), and the concentration of MoS_2_ solution was approximately 2.5 mg/mL. The MoS_2_ solution was bath sonicated for 2 hours in an ultrasonic oscillator (KQ-300DE) to achieve dispersion. Then, the obtained MoS_2_ supernatant solution was centrifuged (TG20) for 45 min at 1500 rpm. A spin coater was used at 3000 rpm for 40 seconds to generate a uniform coating. An inverted microscope (Caikon Reagent Co., Ltd of Shanghai, China) equipped with three magnifications (100, 400, 600 times) was used to observe the interference fringes. The femto-second laser pulse (with a pulse duration of 35 fs and a repetition rate of 2 kHz) used for irradiation was produced by an optical parametric amplifier (TOPAS, USF-UV2), which was pumped by a Ti:sapphire regenerative amplifier system (Spectra-Physics, Spitfire ACE-35F-2KXP Maitai SP and Empower 30).

## Results and Discussion

### Observation and identification of equal-thickness and equal-inclination interference fringes

**Figure 1:**
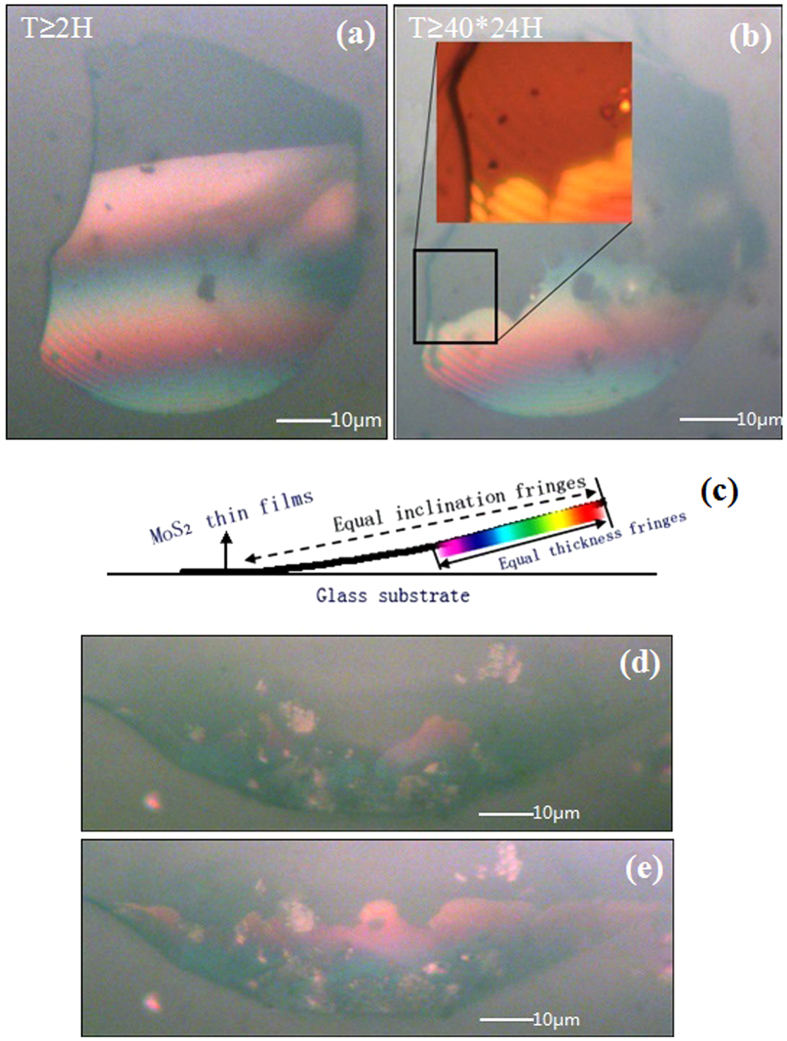
(**a**,**b**) present microscope images of the same piece of MoS_2_ thin films. (**a**) The micrograph of a MoS_2_ thin films newly transferred to the glass substrate. (**b**) The same MoS_2_ thin films after 40 days under the exact same conditions. Inset: enlarged image highlighted by a black square. (**c**) The profile of MoS_2_ thin films. (**d**) The MoS_2_ thin films before irradiation, and (**e**) the MoS_2_ thin films after two hours of laser exposure.

Figure 1a,b are shown micrographs of MoS_2_ thin films newly transferred to the glass substrate (Fig. 1a) and the same MoS_2_ thin films held for 40 days under the exact same conditions (Fig. 1b). The inset in Fig. 1b shows the enlarged image highlighted by a black square.

In Fig. 1a, two sets of fringes—wide, coloured fringes and narrow, black fringes—are observed in the same area on the MoS_2_ thin films with an optical microscope. The wide, coloured fringes result from the equal-thickness interference fringes caused by the reflection of a light beam at the top and bottom surfaces of the wedge-shaped gap between the MoS_2_ thin films and the glass substrate^21^. At the gap of the same height, the straight, coloured fringes are parallel to each other. Additionally, the continuous change of gap height leads to continuous change in the interference colours in the visible spectrum. According to the arrangement of colours in the visible spectrum (where purple corresponds to shorter wavelengths and red corresponds to longer wavelengths), it is inferred that a larger gap exists at the bottom of the thin films. In contrast, the isoclinic interference (narrow, black fringes) is determined by the distances from the upper surface and lower surface of the MoS_2_ thin films, which can be clearly observed if the thickness of MoS_2_ thin films exceeds 250 nm according to the optical theory^21^. Thus, the thickness of the MoS_2_ thin films used here exceeds 250 nm. Further evidence can be found by changing the angle of incident light to the thin films, which causes the isoclinic interference fringes to shift in an obvious and orderly manner. Meanwhile, the wide, coloured fringe resolution changes without displacement, providing additional evidence explaining the equal-thickness interference. Based on the mechanism of isoclinic interference, the homogeneity of the equal-inclination fringes in Fig. 1a indicates the uniform thickness of the MoS_2_ thin films in this work. Additionally, according to the equal-thickness interference theory^21^, the distribution of coloured interference fringes reflects the height variation of the wedge-shaped gap between the MoS_2_ 2D materials and the substrate, which can be used to characterize the dynamic change of that gap.

In Fig. 1b, the wide, coloured fringes have partially disappeared, whereas the change in the narrow, black fringes is negligible after storage of the thin films for 40 days. This phenomenon can be attributed to the decreased gap between the MoS_2_ thin films and glass substrate, which is caused by gravity after storage for 40 days. This results in the disappearance of the equal-thickness interference in the region where the gap height is less than the 1/2 of the minimum visible wavelength. Figure 1c are presented the profile of the MoS_2_ thin films, highlighting the disappearance of the chromatic fringes and the remaining narrow, black fringes.

To further demonstrate that the coloured fringes result from the equal-thickness interference between the upper and lower surfaces of the gap, a sample with a smaller coloured fringe area was chosen and irradiated with an oriented femtosecond laser (wavelength, 700 nm; power, 240 mW). Figure 1d is shown the MoS_2_ thin films before irradiation, and Fig. 1e is shown an image of the same MoS_2_ thin films sample after 2 hours of laser exposure. In the original area in the bottom right, the fringes increase, and red and yellow fringes appear regularly, whereas almost no fringes emerge in the other region. It is speculated that the MoS_2_ thin films are efficiently heated by the femtosecond laser with a certain laser power, causing the air to expand between the thin films and the glass substrate. This leads to a larger wedge-shaped gap because of the flexibility of the MoS_2_ thin films. Hence, the fringes of equal-thickness interference change with the wedge-shaped gap.

By observing the changes in the area with the coloured fringes, the mechanism by which the gap causes the equal-thickness interference fringes is proven and some methods can be demonstrated. For instance, the laser can be used to locally manipulate the gap between the nanosheets and the substrate, increasing the gap from the nanometre scale up to the micron scale. This may lead to the appearance of interference fringes in the heated area. If the angle or height of the gap can be measured and laser-heating or any other method used to manipulate the gap precisely, 2D device fabrication-related research can be performed.

### Angle measurement based on equal-thickness interference fringes

To avoid the interference of equal-inclination interference fringes and minimize the error caused by the forces of the AFM probe, we choose MoS_2_ thin films of moderate thickness (under 250 nm). After being transferred to the glass substrate, the multi-order coloured fringes of equal thickness are observed with an optical microscope at a magnification of 400×. At this point, the medium in the wedge-shaped gap between the MoS_2_ thin films and substrate is air.

**Figure 2:**
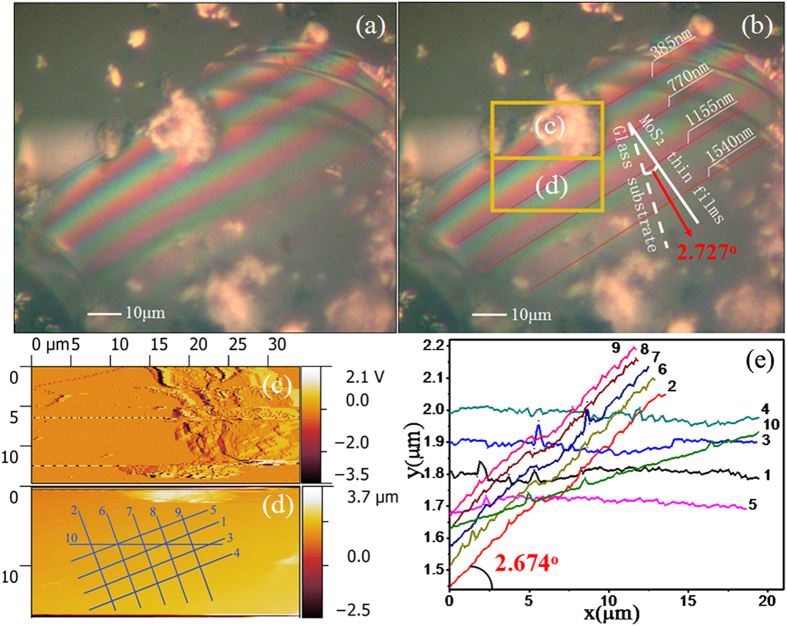
(**a**,**b**) are optical microscope images of the same piece of MoS_2_ thin films. (**c**) The longitudinal amplitude AFM imaging of the upper area, highlighted by the dotted box in (**b**,**d**) the morphological characterization of the lower area, highlighted by the dotted box in (**b**). (**e**) The diagram of relative height curves corresponding to each line, from 1 to 10, marked in (**d**).

Figure 2a is shown one of the MoS_2_ thin films with interference colours. We assume that λ, the wavelength of visible light, is between 350 nm and 770 nm and that the refractive index of the wedge-shaped gap is n_0_ = 1 (vacuum), so that the relative height of the gap (represented as Δh) at specific fringes can be given by Δh = Δk·λ/2n_o_ (Δk = 1, 2, 3….). As shown in Fig. 2b, the junction lines of the red and purple equal-thickness interference fringes are marked with four solid red lines, and the corresponding relative heights calculated with the formula are marked on the appropriate locations. Thus, the distances between the solid red lines can be measured. By solving the arctangent function of the relative height difference versus the relative distances, the angle between the MoS_2_ thin films and the substrate is found to be 2.727° in the direction perpendicular to the equal-thickness interference fringes.

The sample in the AFM image (Fig. 2c,d) is the same as that presented in Fig. 2a. As shown in Fig. 2c, longitudinal amplitude AFM imaging can be used to locate the remarkable characteristic morphology. In Fig. 2d, the height distribution obtained after the morphology characterization is presented. Then, ten sampling curves are plotted in the area; four (1, 3, 4, 5) are parallel to the equal-thickness interference fringes, five (2, 6, 7, 8, 9) are perpendicular to the fringes and one curve (10) is in a direction between those of the others. The corresponding relative height curves are presented in Fig. 2e. It can be seen that the height variation parallel to the interference colours is negligible, whereas the height variation perpendicular to the interference colours is much more obvious. According to the changes in the relative height per unit distance measured by AFM, the direction of the maximum inclination angle of the wedge-shaped gap is approximately 2.674° in Fig. 2e. This measured value is very close to 2.727°, the value calculated by the equal-thickness interference formula, and the error approaches 2%. This result demonstrates the feasibility of computing the wedge-shaped gap angle and relative height in terms of equal-thickness interference fringes in the visible spectrum produced by the interference of visible light. If this method allows both accurate angle and height measurements of nanometre-scale MoS_2_ thin films, it would make the design of micro-scale elastometer and research on nanoscale mechanical properties possible.

### Dynamic bending angle observation with equal-thickness interference fringes

**Figure 3:**
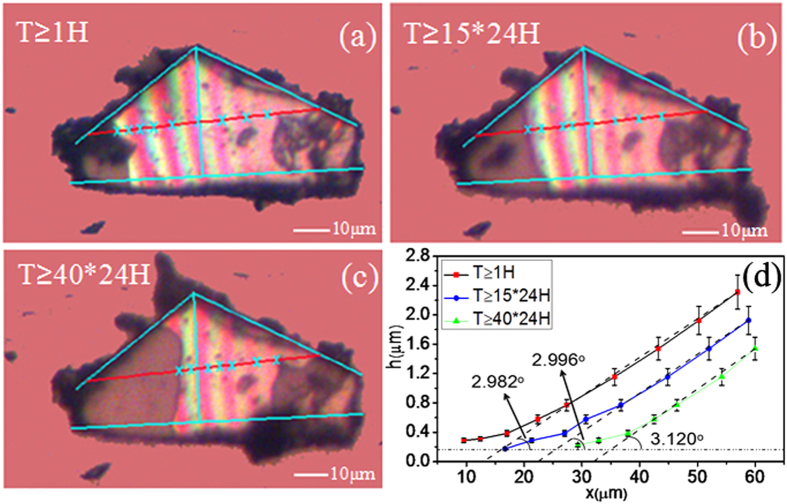
Images of the MoS_2_ thin films with coloured interference fringes on the surface under a microscope after preservation for different times: (a) 1 hour, (b) 15 days, and (c) 40 days. (**d**) The height distribution curves with each height value calculated based on the red horizontal reference line in (**a**–**c**), and the fitting angles at the opening of the wedge-shaped gap are marked at the bottom of the curves.

A sample with thickness ranging from 150 nm to 200 nm was selected to study the mechanical properties and dynamic variation of MoS_2_ thin films. A laser was used to heat the MoS_2_ thin films to obtain equal-thickness fringes with uniform intervals and high contrast colours. The calculated original gap angle of the thin films was approximately 3°, as shown in Fig. 3a. According to the colour variation sequence of the equal-thickness interference fringes, it can be concluded that the opening of the gap is on the right side and that the left side of the gap is smaller. The interference colours are not apparent in the left-side sample because the height of the gap is less than the shortest half-wavelength of the visible spectrum (approximately 175 nm), violet, which does not meet the requirement for equal-thickness interference.

Subsequently, the sample was placed in a dry environment at room temperature for preservation, and observations of the changes in the equal-thickness interference fringes were conducted under the same test conditions. Figure 3b,c are shown the images of the fringes after 15 and 40 days of preservation, respectively. For comparison, blue contour lines and position reference lines are drawn at the same positions in the three patterns. The coloured fringes near the left half of the longitudinal reference line move right, and the area of the MoS_2_ thin films that is free from the interference colours expands significantly. To display the dynamic variation of the gap height resulting from the mechanical properties of MoS_2_, a red transverse reference line is drawn along the long axis of the sample. The heights of the gaps corresponding to several positions (marked with Xs) are calculated according to the equal-thickness interference fringes. Thus, the curves of the height change of the cross-section over time are plotted in Fig. 3d on the basis of the height of the gap at the transverse reference line. The fitting inclination angles between the MoS_2_ thin films and the glass substrate at the gap opening are calculated and marked on the curves. The minimum gap height (approximately 175 nm) able to generate equal-thickness interference is indicated by a horizontal dotted line, as shown in Fig. 3d. The spacing between the MoS_2_ thin films and the substrate decreases at an approximately constant speed with gravitation. After forty days, the gap opening between the MoS_2_ thin films and the substrate is reduced by approximately 30%; nevertheless, the inclination angle at the gap opening remains almost unchanged. The elastic bending deformation occurs under the influence of gravity because of the flexibility of the MoS_2_ thin films. Obvious deformation occurs at the junction of the MoS_2_ thin films and the glass because the greatest gravitational force is experienced by this area; with extended time, the slowly sagging MoS_2_ thin films bend readily at the splitting edge. As a result, the distance between the thin films and the substrate becomes too small to meet the requirement of equal-thickness interference coloured fringes. The fringes in the original area gradually fade, as depicted in Fig. 3a–c (the area on the left side of the longitudinal reference line), whereas the angle approaching the gap opening becomes slightly larger. The three curves in Fig. 3d illustrate the angle changes.

By observing the changes in coloured interference fringes when air is the medium in the wedge-shaped gap, the mechanical properties of the MoS_2_ thin films can be investigated. The gap width between the MoS_2_ thin films and substrate will decrease over time (in 40 days); this is a slow process, and the curvature remains almost unchanged. If minutes or hours are used as the time unit of measurement, the deformation and curvature of MoS_2_ thin films would exhibit great stability without the influence of external forces. Therefore, MoS_2_ thin films have attractive potential applications as elastic devices for the measurement of macro-forces. Laser heating can be used to increase the width of the wedge-shaped gap. However, some other methods may be needed to effectively narrow the gap between the thin films and the substrate if adherence to the substrate surface is required for the 2D materials.

### Utilization of THF liquor to manipulate the dynamic change of curvature of MoS_2_ thin films

To study the dynamic changes in the curvature of MoS_2_ thin films, the films were transferred to the glass substrate after being fully wetted with THF (a volatile polar organic ether), and the displacement of the equal-thickness interference fringes was observed with an optical microscope. For convenient comparison, MoS_2_ thin films with a gap angle of approximately 3° and thickness approximately 150 ~ 200 nm were selected.

**Figure 4:**
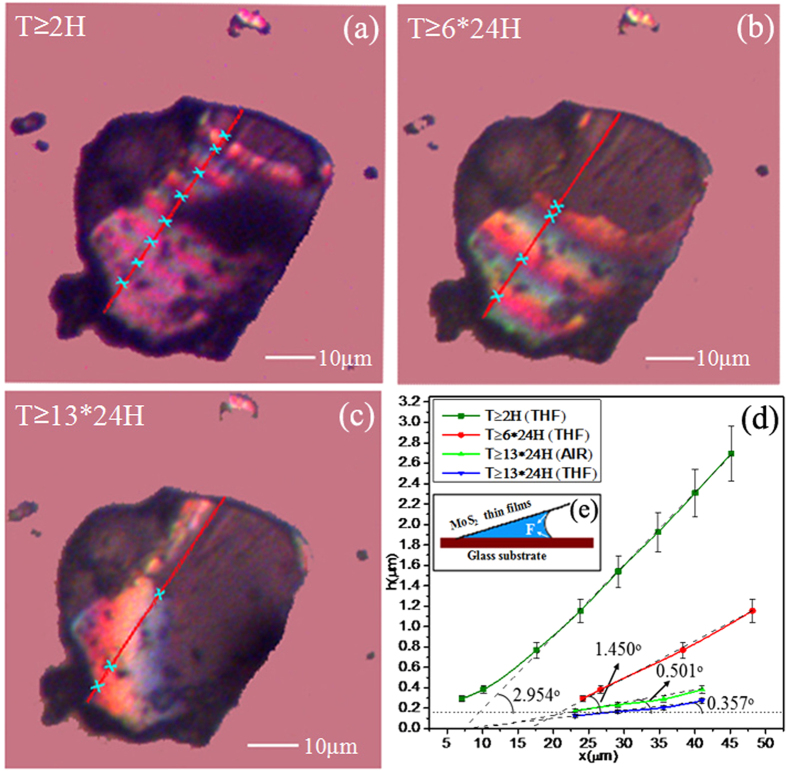
Microscope images obtained after transferring the MoS_2_ thin films to the substrate for (a) 2 hours, (b) 6 days and (c) 13 days. (**d**) The height distribution curves with each height value. (**e**) Schematic diagram of the surface tension of THF filling the gap between the MoS_2_ thin films and the glass substrate.

Figure 4a–c are presented the micrographs taken from the same membrane 2 hours, 6 days and 13 days, respectively, after transfer. Within the relatively short time of 13 days, the total area and number of fringes clearly decrease, whereas the surface area occupied by an individual set of fringes increases significantly. Furthermore, the height profile in Fig. 4d can be calculated by the same method as in Fig. 3. Because of gravitation and the surface tension of the THF, the opening gap between the thin films and the glass substrate is reduced by approximately 87%, and the angle is reduced to 0.357° within 13 days (for convenient comparison, if air is used as the medium, the opening width is reduced by approximately 83%, and the angle at the opening is reduced to 0.501°, as indicated by the light-green line in Fig. 4d).

The explanation for this phenomenon is that the curved liquid surface is formed by THF, which fills the gap between the MoS_**2**_thin films and the substrate. Figure 4e is shown the schematic diagram of the surface tension of the solution, which demonstrates that the surface tension of THF pulls the thin films close to the substrate surface (approximately 10^−6^ N scale), along with simultaneous solvent evaporation. In other words, the angle and width of the gap can be significantly reduced in a relatively short time by introducing the polar volatile solution into the gap between the MoS_2_ thin films and substrate. As a result, the thin films attach to the surface of the substrate smoothly. Additionally, the width of the gap and bending curvature can be significantly reduced in a relatively short time because of a small liquid surface tension. Thus, the bending properties of the MoS_2_ thin films can be studied by using micro-forces.

To further prove that bending curvature can be reduced by applying a polar, low-surface-tension liquid, one other liquids (ethyl alcohol, 95%) was used which has a lower surface tension than THF. After the coloured fringes were obtained from the media, we preserved the membranes for 15 days and calculated the angles between the MoS_2_ thin films and the substrate of different samples after a certain period of time; a portion of the data is shown in Table 1. The larger-surface-tension liquids are found to more obviously reduce the angle compared to the lower-surface-tension liquids.

### Qualitative measurement of the elastic bending properties of MoS_2_ thin films with small forces

**Figure 5:**
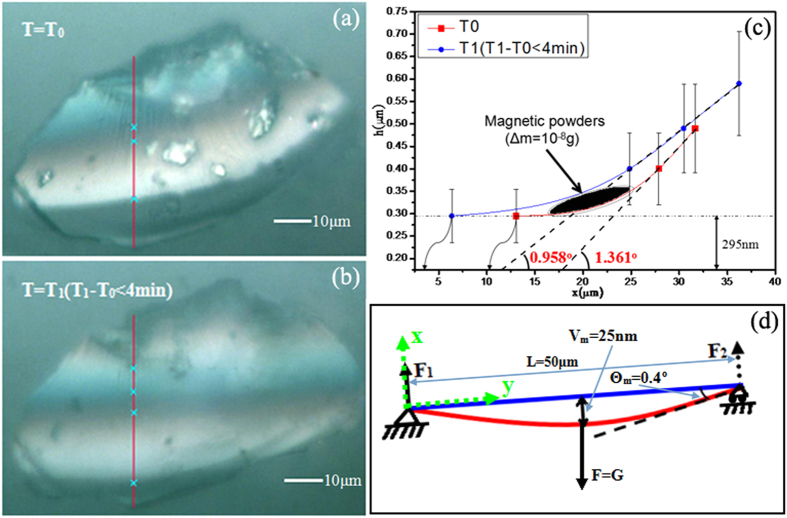
(**a**) The distribution of interference colours after spreading the magnetic powder on the surface of the MoS_2_ thin films at T_0_; (**b**) interference colours after most particles were removed by the magnetic force within 4 minutes; (**c**) relative height-position curves plotted based on relative heights determined according to the distribution of interference coloured fringes in (**a**,**b**) and the fitting curvature of the MoS_2_ thin films; and (**d**) schematic diagram of the “simple beam” model. The left end is fixed, while the right end can move freely.

A thin layer of magnetic powder (the main ingredient of which is ferroferricoxide) was spread on the surface of a selected MoS_2_ thin film with interference colours. The size of each tiny particle attached to the thin film surface was less than 10 μm. If the density of the ferroferricoxide is 5.18 g/cm^3^, the amplitude of the mass of each particle is approximately 10^−11^ kg. That is, the total gravity of all particles applied to the MoS_2_ thin films is on the scale of 10^−9^ N. Then, the coloured fringes change rapidly when an external magnetic field is applied to the system and removed, reflecting the change in the bending curvature of the thin films. Figure 5a is presented an image of a MoS_2_ thin film whose wedge-shaped gap with the substrate is reduced but whose curvature increases when the magnetic particles are placed on the surface of the thin films. After 4 minutes, the thin films rebound because of their resilience when most particles are removed by the magnetic force (Fig. 5b). The relative height-position curves corresponding to the red reference lines in both photos are plotted according to the positions of the interference colours, which correspond to the relative heights in Fig. 5a,b. Both of the approximate curves are depicted in the schematic shown in Fig. 5c, which demonstrates the elastic deformation of the membrane that occurs during the removal of the particles. Compared with T_0_, the maximum gap between the thin films and the substrate increases by approximately 25 nm at T_1_. The curvature decreases by approximately 0.403° because of the resilience of the thin films.

To qualitatively study the elastic bending properties of the thin films, the deformations of the thin films are regarded as 1D “simple beams” before and after gravity is applied to the powder^22^, as represented in Fig. 5d. In this model, the plane of the 2D thin films is equivalent to a segment with the length L. The red and blue curved lines represent the bent thin films under the gravitational force of the magnetic particles and the flat membrane free from the external forces, respectively. As depicted in the schematic diagram of the “simple beam” model, the left end is fixed, while the right can move freely. F_1_ and F_2_ are normal forces acting on the nanosheets. “Deflection” represents the linear displacement of the centroid of the section in the vertical direction, and both the maximum deflection V_m_ and the maximum rotational angle θ_m_ are marked in the schematic diagram. As for forces in the practical situation, the normal force from the substrate acting on the thin films is equal to the magnitude of F_1_ at the fixed end, and the other tension from the deformation-free part to deformation part of the thin films is approximately equal to F_2_.

The differential equation of the deflection curve is defined as:


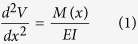


where E is Young’s modulus-like for the MoS_2_ thin films of a particular shape, I is the product of inertia, and M(x) is the bending moment of the distortion of the material somewhere along the cross-section. V is the deflection, θ is the rotational angle, and the relationship between θ and V can be written as:


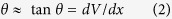


The effect of the gravitational force of the magnetic particles is approximately equivalent to G; thus, for both relations of θ and x, V and x can be solved, and x is a location along the line. If we suppose that the approximate location at which the force acts is the midpoint of the thin films, the maximal deflection V_m_ and rotational angle θ_m_ could be given as:


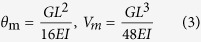


The spring constant of the single beam is defined by k_y_ = F_y_/V_m_ and, hence, given by:


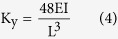


Thus, the spring constant for MoS_2_ thin films can be theoretically obtained according to (4). If the membrane in Fig. 5a,b can be regarded as an isosceles trapezoid, by substituting the calculated product of inertia into equation (3), with other known measurable values (G, L, θ_m_ and V_m_), we can obtain E, the Young’s modulus-like value, which is on the order of 100 Pa. E is described as Young’s modulus-like and has a similar physical meaning to the elastic modulus. E can be used to describe the tendency to recover after withdrawing forces from the MoS_2_ thin films. The value of the Young’s modulus-like E calculated by this method is much smaller than the Young’s modulus of the MoS_2_ thin films obtained from the nanoindentation experiments^8^,^10^. It is supposed that the MoS_2_ thin films in this research only contact the substrate at one end, that the other is free and that the MoS_2_ used in nanoindentation is fixed at both ends. Additionally, the magnitude of the distance between the point at which the force acts and the fixed end is on the micron scale in our experiment, whereas it is on the nanometre scale in the nanoindentation experiments. In addition, the elastic properties of 2D materials of different shapes are not the same. The MoS_2_ thin films in our research are relatively sensitive to small forces. Thus, this material would be appropriate for the fabrication of micro-force sensors because of its readily observable interference colours.

## Conclusions

Coloured equal-thickness interference fringes observed with an optical microscope were used to calculate the curvature of MoS_2_ thin films, and the error in the measurement was determined by AFM to be less than 2%. In the absence of external influence, the bending curvature of the MoS_2_ thin films tended to be unchanged after forty days of preservation, exhibiting excellent stability. After pulsed laser irradiation, the bending curvature of the MoS_2_ thin films increased within 2 hours, and a polar solvent could be used to reduce the bending curvature because of the liquid surface tension. The MoS_2_ thin films deformed rapidly on the minute-scale immediately after the application of a small force to the surface, and the Young’s modulus-like value was obtained. It is theoretically possible to fabricate micro-Newton or nano-Newton micro-elastic force sensors with MoS_2_ thin films. This research provides a non-contact method for observing the elastic bending properties of a 2D material with an ordinary optical microscope, and characterizes the mechanical properties of 2D materials. This work also brings a new idea and approach for small force detection based on the excellent elastic properties of 2D materials.

## Additional Information

**How to cite this article**: Wang, P. *et al.* Investigation of the dynamic bending properties of MoS2 thin films by interference colours. *Sci. Rep.* **5**, 18441; doi: 10.1038/srep18441 (2015).

## Figures and Tables

**Table 1:** The different angles between the MoS_2_ thin films and the substrate at different times with tetrahydrofuran (THF)and ET(ethyl alcohol).

Liquid	Surface tension coefficient (mN/m)	θ_0_ (T ≥ 2H)	θ_1_ (T ≥ 6*24H)	Δθ_1_ (ΔT ≥ 6*24H)	θ_2_ (T ≥ 13*24H)	Δθ_2_ (ΔT ≥ 13*24H)
THF(25 °C)	26.4	2.954°	1.450°	1.604°	0.357°	2.579°
ET (95%, 25 °C)	22.88	3.340°	2.882°	0.458°	2.571°	0.769°
